# Feasibility of Big Data Analytics to Assess Personality Based on Voice Analysis

**DOI:** 10.3390/s24227151

**Published:** 2024-11-07

**Authors:** Víctor J. Rubio, David Aguado, Doroteo T. Toledano, María Pilar Fernández-Gallego

**Affiliations:** 1 School of Psychology, Universidad Autónoma de Madrid, 28049 Madrid, Spain; david.aguado@iic.uam.es; 2 Instituto de Ingeniería del Conocimiento, 28049 Madrid, Spain; 3 Polytechnic School of Engineering, Universidad Autónoma de Madrid, 28049 Madrid, Spain; doroteo.torre@uam.es (D.T.T.); mariapilar.fernandezg@estudiante.uam.es (M.P.F.-G.)

**Keywords:** personality assessment, voice analysis, externalization of personality

## Abstract

(1) Background: As far back as the 1930s, it was already thought that gestures, clothing, speech, posture, and gait could express an individual’s personality. Different research programs, some focused on linguistic cues, were launched, though results were inconsistent. The development of new speech analysis technology and the generalization of big data analysis have created an opportunity to test the predictive power of voice features on personality dimensions. This study aims to explore the feasibility of an automatic personality assessment system in the context of personnel selection. (2) Methods: One hundred participants were recorded during an individual interview for voice analysis. They also completed the NEO-FFI and were required to ask and collect the assessment of their personality by a close significant other. Furthermore, an expert estimated participants’ personality dimensions based on the viewing of the recorded interviews. (3) Results: Results showed there are specific voice features related to the externalization of individuals’ personalities (predictions ranging from 0.3 to 0.4). Voice features also predicted significant others’ estimations and expert ratings of the target individual’s personality, though the features were not exactly the same. (4) Conclusions: It is noteworthy that predictions were made based on voice recordings obtained using ordinary devices in controlled but not restricted speech situations, which may make such an approach a promising tool for personality assessment in contexts such as personnel selection.

## 1. Introduction

People often make assumptions about others’ internal characteristics, even when there is minimal or no prior acquaintance [1]. Research on interpersonal and social perception has shown a notable correlation between how people rate others’ personalities and how they rate their own [2]. Ambady and Rosenthal’s [3] meta-analysis found an overall accuracy of 0.39 in predicting various objective outcomes, even in zero-acquaintance situations. This suggests that individuals may communicate something about their personalities that can be interpreted by observers [4,5].

Various research programs have explored the nature of the cues involved in spontaneous trait inference, with some focusing specifically on linguistic signals. The idea that personality can be revealed through the voice is likely as old as human language itself, and it has long been used to form judgments about others. Allport and Cantril [6] were among the first scholars to conduct a systematic analysis of the relationship between speech and personality. They carried out 10 experiments to determine whether the voice could serve as a valid indicator of personality. They concluded that (1) raters were highly successful in matching information about 12 distinct personality traits to the corresponding voices; (2) there was strong interrater agreement, although this agreement was not always accurate; and (3) personality attributions were more accurate when based on a summary of speech characteristics rather than on a single trait.

Since then, numerous studies have examined the relationship between speech and personality, all of them building on Polzehl’s observation that “all voices leave an impression” [7] (p. 23). Initially, the results were inconclusive. For instance, Sherer [8] proposed an adaptation of Brunswick’s [9] lens model to structure the components involved in personality judgments from voice and speech. According to this model, personality traits are ‘externalized’ through objectively measurable speech variables that act as ‘distal cues’. These cues are perceived by listeners and represented as ‘proximal cues’, which form the basis of the personality attributions made by observers. Sherer analyzed four aspects of the ‘externalization of language’: vocal aspects of the voice (frequency, intensity, and quality), fluency of speech (including pauses and discontinuities), morphological and syntactic aspects, and conversational behavior. Through this analysis, Sherer remained skeptical about the results of prior studies, though he did find associations between fundamental frequency, intensity, and traits like competence and dominance, which were linked to Extraversion.

Brown and Bradshaw [10] conducted another review that focused on three facets: accuracy, referring to how well judges could identify personality traits from a speaker’s voice; externalization, concerning the relationship between voice parameters (e.g., frequency, intensity) and personality traits; and attribution, examining how variations in speech features influence personality judgments. Regarding accuracy, they noted that the lack of objective personality measures in earlier studies hindered conclusive results, unlike the accuracy found in judgments of more easily measurable traits such as age, sex, or social class. For externalization, they pointed out difficulties in measuring speech and personality. However, attribution studies proved more fruitful, highlighting the role of speech rate, pauses, and temporal patterns in personality judgments. For example, Addington [11] found correlations between traits like Extraversion and features such as speaking speed and pitch variation. Aronovich also identified correlations between vocal features like intensity variation, perceived pitch, and traits like self-confidence or submissiveness/dominance [12].

Furnham [13] reviewed studies analyzing speech variables and personality traits, particularly Extraversion. He criticized the methodological issues in many studies and the lack of theory-driven approaches but still noted relationships between Extraversion and voice intensity and quality. He also referred to the main concerns related to the lack of a universal acceptance of a common personality structure as well as a shared approach to personality measurement.

More recently, advances in speech analysis technology, on the one hand, and the use of widely accepted Big Five theoretical personality framework and its measurement instruments, on the other, have produced clearer findings. For instance, prosodic features (e.g., pitch and frequency variation) have been linked to Extraversion [14], and speech frequencies in the Chinese language were correlated with psychoticism, Extraversion, and Neuroticism [15]. McAlleer and colleagues found that short utterances can predict personality, with the f0 trajectory (intonation) strongly associated with perceived trustworthiness [16,17]. Polzehl and colleagues conducted experiments that successfully classified individuals with high and low levels of traits like Neuroticism and Extraversion using voice features often employed in emotion recognition [7,18,19]. Stern et al. [20] found substantial negative correlations between voice pitch and traits like sociosexuality, dominance, and Extraversion, suggesting that vocal characteristics can partially reflect personality, particularly in self-reports of these traits. Marrero et al. [21] expanded on this by examining how voice samples can predict self-reported personality traits using machine learning. They found that vocal features could account for about 10% of the variance of personality traits, also showing promising results in predicting other characteristics such as depression. Additionally, Schmitt et al. [22] emphasized the role of auditory cues in designing voice-based interfaces, which can influence user behavior and perceptions through strategies like personification and contextualization, pointing out the potential applications in human–computer interactions. Likewise, Van Zant and Berger [23] demonstrated that paralinguistic cues such as pitch and volume modulation can enhance persuasive communication by signaling confidence, further illustrating the persuasive power of vocal demeanor beyond the content of speech.

Summing this up, Breil et al. [24] reviewed 32 studies that have used up to 39 non-verbal cues, including paralinguistic cues, and established that some of them showed significant correlations with Big Five traits. Specifically, with regard to expressive/varying voice, fluent speaking, pleasantness of voice, powerful/confident voice (vs. soft), loudness, speech rate, and speech vs. non-speech correlated with Extraversion, all of them but powerful/confident voice and speech rate correlated with neuroticism; pleasantness of voice correlated with openness; and fluent speaking correlated with Agreeableness. Collectively, these studies suggest that vocal characteristics not only serve as valid cues to personality traits but also play a broader role in shaping user experiences and influencing interpersonal and persuasive communication.

Despite these advances, some limitations persist, particularly regarding the artificial conditions under which speech is usually produced in these studies. For example, Gocsál analyzed female listeners’ judgments of male speakers based on spontaneous speech and found that temporal parameters were correlated with inferred openness and Extraversion [25]. However, unlike previous findings, fundamental frequency did not correlate with any inferred personality traits, suggesting that in spontaneous speech, other factors may overshadow the role of frequency observed in isolated speech or text readings.

One of the main challenges is to develop a user-friendly reliable system for assessing personality traits based on speech. Advances in computing power, big data analysis, and accessible technology have created opportunities for automatic personality assessment. Additionally, the development of robotics has spurred interest in identifying vocal characteristics to generate artificial personalities [26].

It has been argued that the limitations in studying nonverbal cues of personality, particularly speech features, stem from a lack of collaboration between two fields: psychological research on personality judgments and artificial intelligence research on personality computing [27]. This study aims to bridge that gap. By building on the framework suggested by Cannata and colleagues, the current research explores the feasibility of using natural speech settings to extract and analyze vocal characteristics and harmonize nonverbal features. In line with these authors, a combination of different personality measures should be used as the gold standard, integrating self-reports, informant reports, and behavioral assessments.

### Voice Analysis for Paralinguistic Information Extraction

From a technological perspective, voice analysis has proven effective in extracting not only the linguistic content of speech but also a range of information unrelated to the words themselves. One notable example is Automatic Speaker Recognition, which has been in development since at least 1996 and can accurately identify speakers [28,29,30]. This technology relies on short-term spectral features known as Mel-Frequency Cepstral Coefficients (MFCC), which summarize the energy distribution of audio over 20–40 ms intervals on a psychoacoustically derived frequency scale (the Mel scale).

Furthermore, it is well-established that certain medical conditions affecting the voice production system can also be detected through voice analysis. Many neurological conditions, including dementia, Alzheimer’s, and Parkinson’s disease, have early detectable effects on speech. Non-automated diagnostic procedures often include tasks where patients speak to observe these effects. Automated procedures have also been proposed for the early detection of such conditions. For example, Satt [31,32] used speech duration features and measures of regularity to detect early-stage dementia. Similarly, Weiner et al. [33] used speech/silence duration and word duration features to identify early signs of Alzheimer’s disease. Skodda and Schlegel [34] observed that speech in Parkinson’s patients exhibited rhythmic alterations, including increased articulation rates and fewer pauses, while Rusz et al. [35] identified differences in articulation, phonation, and prosody.

Voice analysis has also been proposed for detecting more dynamic internal states, such as fatigue, sleep deprivation, and emotions. For instance, Baykaner et al. used MFCC, autocorrelation coefficients (ACCs), and energy contour features to predict sleep deprivation times (in minutes) with an error margin between 5% and 12% and to assess mental fatigue with correlations above 0.69. This study was conducted in the context of astronaut training [36].

Emotion detection, another major focus in paralinguistic studies, has garnered significant attention. Emotions play a crucial role in speech communication and are thus important in human–machine interaction systems. Moreover, studies have found a relationship between the accuracy of emotion detection and personality judgments in humans, suggesting that detecting emotional states may provide valuable insights into personality [37]. In 2011, Schuller et al. [38] proposed a feature set of 1941 audio-related features composed of 25 energy and spectral low-level descriptors (LLD) multiplied by 42 functionals, 6 voicing-related LLDs multiplied by 32 functionals, 25 delta coefficients of energy/spectral LLDs multiplied by 23 functionals, 3 delta coefficients of voicing-related LLDs multiplied by 19 functionals, and 10 voiced/unvoiced duration features. This set has been used successfully in various challenges, such as the Audio Visual Emotion Challenge (AVEC) 2011, for detecting emotions from speech. These features are also easily extractable using the open-source software OpenSMILE version 2.3 [39], which will be used in the current study.

In summary, automatic voice analysis has received considerable attention in paralinguistic studies, particularly for detecting both physical conditions, such as disease, and internal psychological states, like emotions. Given this, it can also be argued that voice analysis could be effectively used to assess individuals’ personality traits. Indeed, several international challenges have already addressed the estimation of personality traits from voice and video. One of the earliest was the Interspeech 2012 Speaker Trait Challenge [40], which included a subtask for identifying the Big Five personality traits from speech using the Speaker Personality Corpus [41]. The goal was to determine whether these traits were in the upper or lower part of the distribution based on judgments from 11 raters. The audio content consisted of 10-s excerpts from French news bulletins. This challenge also used OpenSMILE for feature extraction, similar to the approach in this study, but employed over 6000 features.

In recent years, other challenges have focused on personality trait estimation. These include the ChaLearn Looking at People 2016 First Impressions Challenge [42], held during the European Conference on Computer Vision (ECCV) 2016, and the ChaLearn Looking at People—Job Candidate Screening Coopetition [43], organized during the CVPR 2017 conference. Both challenges used the First Impressions V2 Corpus [44], which consists of 10,000 video segments (15 s each) extracted from YouTube videos, where speakers face the camera to simulate a multimedia CV. The corpus was labeled using Amazon Mechanical Turk. In the 2016 challenge, participants were tasked with determining the Big Five personality traits, while the 2017 edition focused on providing overall recommendations about job candidates and explaining those decisions.

Building on these efforts, the ChaLearn Looking at People Challenge [45] in 2021 introduced a new dataset (UDIVA) [46] for estimating personality traits based on dyads and small group interactions, expanding the scope to more natural social settings. In addition, the recently published Multimodal Personality Traits Assessment (MuPTA) Corpus [47] added further depth by offering a balanced sample of Russian speakers, incorporating both spontaneous and scripted speech. Importantly, the development of open-source tools such as OCEAN-AI [48] has made it possible to integrate multimodal data (audio, video, and text) to predict personality traits, marking a significant advancement in the field. These tools are key for furthering the understanding of how speech patterns relate to personality.

Some of the latest pieces of research published in the field of automated video interview personality assessment have demonstrated that audio signals can be effectively used to predict both self-reported and expert-rated personality traits [49,50].

By assuming that speech reveals ‘ourselves’, as some personality theorists advocate [6,51], and given that current voice analysis technology has shown to be useful in inferring different internal states and traits, including personality dimensions, this paper presents an exploratory study to test the feasibility of an automatic assessment of personality based on big data analysis of voice processing using situational tests in natural settings. As previous studies have shown, there are voice features related to the attribution of different personality traits that are useful for classifying targets according to them. Nevertheless, many of those studies collected speeches in non-natural settings and/or used actors, and nearly none of the previous studies focused on long interviews simulating a real job recruiting interview in a realistic setting. Moreover, a key aspect in exploring the accuracy of personality judgments based on voice features is the criterion used for its testing [10]. Many studies have used rating scales and first impression judgments of targets’ personality traits instead of self-reported personality trait assessment, as well as targets’ personality ratings reported by close others and third-party experts. Furthermore, as some authors posed [7], just slight changes in voice parameters may affect the perception of the target’s personality, which may affect the consistency of estimations.

Therefore, we will test whether the automatic voice analysis of data gathered in a weakly controlled assessment situation can effectively and accurately estimate the Big Five personality dimensions of individuals. We have focused on two of the facets highlighted by Brown and Bradshaw [10]: (1) the externalization, regarding the relationships between voice features, i.e., frequency, intensity, etc., and personality characteristics and (2) the accuracy, referring to the extent to which judges can identify personality traits from a speaker’s voice. Therefore, not only will the individuals’ self-report be collected but also the close-others judgments and the expert zero-acquaintance ratings of target individuals’ personality traits.

In particular, the following questions are addressed:

1. Are there voice features related to the self-reported Big Five personality scores of individuals? In other words, are there specific voice features related to the externalization of individuals’ personality traits? It is expected, given the previous results analyzed, that personality is expressed by speech and voice markers, and these might be useful predictors of personality;
2. Are voice features related to externalization the same as those related to the accuracy of personality judgments? Put another way, are voice features related to the close-others personality judgments and expert zero-acquaintance ratings the same as those that predict self-reported personality? It is expected that the expressive features would not be exactly the same as those used by others to make personality judgments;
3. Can we design a feasible assessment setting that could be useful for an automatic personality assessment procedure? In other words, can we predict personality dimensions based on voice recordings obtained by ordinary devices in controlled, but not restricted, natural speech situations? It is expected that if voice features predict personality dimensions, these features can be processed and used in non-experimental conditions and, therefore, implemented in ordinary assessment contexts such as Asynchronous Video Interviews (AVIs).

## 2. Materials and Methods

### 2.1. Participants

One hundred females voluntarily participated in the study. Participants were divided into two samples. Sample 1 consisted of 78 female psychology students aged from 18 to 27 years old (Mean age = 20.18). Sample 2 consisted of 44 female participants aged from 18 to 54 years old (Mean age = 29.42). We used two samples to explore the consistency of our findings. Due to the large number of features used as voice markers, the probability of finding spurious correlations was high. Therefore, the use of two different samples helped us to control this effect.

All participants were recruited from the first author’s School of Psychology participants’ pool of students, where they can register for an extra credit. The institutional system facilitates the recruitment, schedule, and assignment of credits to students interested in participating in psychological research. The rest of the students are provided with equitable alternative activities. Due to the exploratory nature of our study, the sample size was not based on an explicit power analysis. Moreover, the characteristics of the study (including individual interviews) and the availability of the participants constrained the recruitment of larger samples. However, based on a power analysis aiming at 80%, a sample size of N = 100 detects the effects of correlations of about 0.2, considered enough for the aims of this study.

### 2.2. Measures

#### 2.2.1. Voice Features

In this study, we used the same feature set as the one proposed in the Audio Visual Emotion Challenge (AVEC) 2011 to detect emotions. These features have been obtained from the audio using the open-source software OpenSMILE [39].

The feature set consists of 1941 features, composed of 25 energy and spectral-related low-level descriptors (LLD) (loudness, zero crossing rate, energy in bands from 250–650 Hz, 1–4 kHz, 25%, 50%, 75%, and 90% spectral roll-off points, spectral flux, entropy, variance, skewness, kurtosis, psychoacoustic sharpness, harmonicity and MFCC 1–10) with 42 functionals, 6 voicing related low-level descriptors (F0, probability of voicing, jitter, shimmer (local), jitter (delta: “jitter of jitter”), logarithmic Harmonics-to-Noise ratio (logHNR) with 32 functionals, 25 delta coefficients of the energy/spectral low-level descriptors with 23 functionals, 6 delta coefficients of the voicing related low-level descriptors with 19 functionals, and 10 voiced/unvoiced durational features. The Low-Level Descriptors and the functionals are summarized in Table 1 and Table 2.

#### 2.2.2. Personality Assessment

The Big Five self-report personality assessment was obtained from the NEO-FFI scores [35].

The judgments of the close others regarding the target individuals’ personality were obtained using a 3-point (1-low, 2-medium, 3-high) 5-dimension rubric that each relative or close friend completed. The rubric included a brief description of each of the Big Five dimensions and levels, i.e., Neuroticism, Extraversion, Agreeableness, Openness to Experience, and Conscientiousness. The rubric was an adaptation of the summary sheet provided by the manual of the Spanish adaptation of NEO-FFI [52]. The rubric is included in the Supplementary Materials.

Expert ratings of the target individuals’ personality traits were appraised after watching the complete video recording of each participant. Personality dimensions were assessed using a 15 cm visual analog scale and according to a rubric describing the behavioral cues of each trait. The score corresponded with the distance in centimeters from the 0 cm mark to the expert’s indicated mark. The assessment form is available in the Supplementary Materials. Two highly experienced experts participated in the assessment of the video recordings. Expert 1 is a female Ph.D. and Expert 2 is a male Ph.D., both with over 25 years of expertise in assessing personality and other psychological variables through interviews. Before the assessment, the experts conducted an inter-rater agreement calibration process to ensure consistency in their evaluations. This process continued until they achieved 100% agreement on the key cues used for personality judgments. Following this calibration, both experts rated a subset of the videos (N = 20) to estimate the reliability of their evaluations using the intraclass correlation coefficient (ICC). The results showed moderate to strong agreement for the dimensions of Emotional Adjustment (ICC = 0.70), Extraversion (ICC = 0.73), Agreeableness (ICC = 0.74), and Conscientiousness (ICC = 0.82). However, the ICC for Openness to Experience was lower (0.23). Experts discussed disagreements, rated the videos again to increase Openness to Experience’s inter-rater agreement and, after that, the remaining videos were assessed by Expert 2.

### 2.3. Procedure

After obtaining the approval of the Institutional Review Board, the participants were called to a first group session in which they were informed about the aim of the research and signed the informed consent. In that session, the participants fulfilled the 60 items of the Spanish adaptation of NEO-FFI [52], including the Big Five dimensions. They were also appointed to an individual interview, which took place in the following two weeks. In addition, they were required to ask and collect the assessment of their personality made by a close relative or friend using a rubric and bring it to the individual session.

Each session took about 7 min. on average and was audio and videotaped. The audio recording was used later to obtain the voice features, as explained below. The videotape recording was watched by one of two experts in personality assessment and used to estimate the participant’s personality dimensions.

The audio files were recorded in a high-quality format using the WAV format with a 16 kHz sampling rate, single channel, and 16 bits per sample. The sessions were recorded in a usually quiet office, though some recordings captured some noise originating from outside the room. The video files were recorded with an HD commercial camera.

The interview was aimed at eliciting the verbal production of participants, and it included warm-up questions such as “What’s your name?”, “What are the courses you are enrolled in?”, “How many courses have you already passed?”. Afterward, participants were asked about themselves: “Please, tell me about you, your hobbies and interests”, “Why did you choose what to study?”, “How are you guiding your career development?”, “Please describe how you think the world in general, and specifically your life, is going to be in 15 years”. Finally, the participants were thanked for their contribution, and the session was finished.

Since the interview included both open-ended and more structured questions, allowing very brief answers, we only used the answers to the open questions where a more spontaneous speech could be produced. We manually selected the audio segments corresponding to these answers. Each audio selection had a different length, ranging from 15 min to 49 s (Mean length = 172.8 s; SD = 102 s). In total, we processed 4 h and 48 min of audio corresponding to 100 different participants.

### 2.4. Data Analysis

We carried out two types of analysis. The first one explored the correlations between the characteristics of the voice and the estimation of personality made through the questionnaire. For this, the consistency between the correlations found in sample 1 and sample 2 was explored. The second type of analysis was predictive. Different models were used to predict personality traits from voice characteristics. All analyses were performed in Python using the Pandas, SciPy, and Scikit-learn libraries.

## 3. Results

### 3.1. Correlation Analysis

The correlations between the Big Five self-reported dimensions and the estimations from the close others and the experts are shown in Table 3. This table shows that there were consistent (low to moderate) correlations between the significant others and the target individual’s self-report. All but Openness showed significant correlations, ranging from 0.32—Neuroticism to 0.51—Extraversion. On the contrary, the correlations between the expert scores and individuals’ self-assessments were significant in only two cases: Responsibility (0.34) and Extraversion (0.31). As expected, these two dimensions showed significant correlations between the judgments made by the close others and the expert’s scores.

The correlations between voice features and personality dimensions are shown in Table 4. Given that the number of features extracted from the audio is rather large, our first analysis tried to detect those directly related to the externalization of personality. We conducted a correlation analysis between all the 1941 audio features and the values for each of the personality dimensions, i.e., Neuroticism, Extraversion, Openness, Agreeableness, and Conscientiousness, which had been obtained by self-report, close-others judgments, and expert ratings. Table 4 shows the features that presented the higher correlations.

All the correlations shown were moderate but statistically significant (*p* < 0.001), with a maximum of 0.50. The audio features that were found to show the highest correlation with each personality dimension (Table 4) are described in descending order of magnitude in the next subsections.

#### 3.1.1. Voice Features Related to Extraversion

The highest correlations between voice features and personality scores were found for Extraversion. When considering the self-reported Extraversion scores, the voice feature with the highest correlation (0.47) is mfcc_sma[3]_lpgain. This feature is derived from the third MFCC coefficient, which compares the amount of energy in different frequency bands. This feature was obtained by first smoothing it with a smoothing moving average filter (sma) and then computing the linear prediction gain (lpgain) over the total audio of the speaker. A high linear prediction gain means that the value to predict is mainly predicted by the past samples. Essentially, the feature measures the predictability of the energy in different frequency bands based on recent past values, which is higher when high-energy phones such as vowels are long and have high energy.

When considering the personality scores estimated by the expert, Extraversion was also the trait with the highest correlation with voice features, up to 0.48. The feature with the highest correlation is related to the range of variations (interquartile range 2–3) of the smoothed (sma) magnitude (measured with an L1 norm) of the auditory spectrogram (audspec). The higher this feature, the greater the variation in loudness, which seems to indicate that the larger the range of variations in voice volume, the higher the levels of Extraversion attributed.

The correlation of Extraversion, as measured by the close others and acoustic features, was lower (0.40). Extraversion measured by close others does not present the largest correlation among the personality dimensions, as found for the self-reported and expert-reported cases, but is still very close to the highest correlations, with scores provided by close others (0.41). The correlation was observed with a different feature: the dispersion of the lower values (interquartile range 1–3) in the variation in the variations (delta) of the soothed (sma) voicing estimation (voicing-FinalUnclipped). In this case, the correlation is not as easy to interpret because voicing depends on the type of phone uttered. The feature could be related to how varied the changes from a voiced to an unvoiced sound and vice-versa are, which could be related to the variations in the speaking rate.

#### 3.1.2. Voice Features Related to Neuroticism

We found that Neuroticism shows a high correlation with voice features, particularly for expert-provided personality scores. Specifically, we found a high correlation (−0.47) between this personality trait and the first quartile of the smoothed (sma) spectral magnitude roll-off (pcm_Mag_spectralRollOff75.0). The spectral magnitude roll-off is the frequency that divides the energy in the spectrum so that 75% of the energy is below that frequency. Given that the correlation is negative, Neuroticism is associated with many low values of this parameter, indicating that most energy is concentrated in low energies and could be related to very low volume in the voice.

When considering self-reported personality scores or scores provided by close others, correlations are lower (0.37 and 0.32, respectively). For self-reported personality scores, the highest correlation was with the first percentile of the variations (delta) of the smoothed (sma) voicing probability (voicing—FinalUnclipped). A high value of this feature indicates that the smallest variations in the voicing probability tend to be relatively large, which could indicate instabilities in the production of voiced speech. For personality scores provided by close others, the highest correlation is found with the minimum value of the range of variations (minRangeRel) of the third MFCC coefficient, which compares the amount of energy in different frequency bands. Therefore, given that the correlation is positive, Neuroticism could be associated with higher values of this parameter, indicating a particular distribution of energy in the spectrum, similar to the findings with the expert-provided personality scores.

#### 3.1.3. Voice Features Related to Openness

The highest correlation between Openness and voice features was −0.41, found between close other’s reported Openness and the percentage of time (upleveltime50) that the variation (delta) of the smoothed (sma) fundamental frequency or pitch (F0final) was above the minimum + 50% of the range of variation. Given the negative sign of the correlation, it seems that it indicates that Openness is related to having faster increases in pitch and slower decreases.

When considering self-reported Openness, the correlation with the percentage of time (upleveltime25) that the variation (delta) in the smoothed (sma) magnitude (lengthL1norm) of the auditory spectrum is above the minimum + 25% of the range of variation was smaller (0.36). In this case, the correlation is positive, indicating that Openness is related to having most of the time significant variations in the volume of the voice.

Finally, the Openness scores provided by experts showed the highest correlation (−0.36) with a feature that is difficult to interpret (mfcc_sma[10]_lpc3). This is the 3rd linear prediction coefficient (which relates the present sample with the value of three samples in the past) of the 10th MFCC Coefficient, which depends on a complex distribution of energy across different frequency bands.

#### 3.1.4. Voice Features Related to Agreeableness

The largest correlation found between the Agreeableness reported by close-others and voice features was −0.41 with mfcc_sma[5]_risetime. This feature represents the percentage of time that the fifth MFCC Coefficient is rising, but this coefficient depends in a complex way on the distribution of energy in different frequency bands, making it difficult to interpret.

When considering personality scores provided by experts, the highest correlation (0.35) was with pcm_Mag_spectralRollOff75.0_sma_linregc1. This feature is the slope of the linear regression of the smoothed spectral roll-off. The spectral magnitude roll-off is the frequency that divides the energy in the spectrum so that 75% of the energy is below that frequency. A high value of this coefficient indicates a steeply rising slope of this frequency, meaning fast increases in frequency.

Finally, when considering self-reported personality scores, the highest correlation for the Agreeableness trait (−0.33) was with audspec_lengthL1norm_sma_de_meanSegLen, which represents the mean segment length of the variations in the (smoothed) magnitude of the auditory spectrum. Since the correlation is negative, Agreeableness seems to be related to frequent changes in the magnitude of the spectrogram, which could be related to a more varied voice.

#### 3.1.5. Voice Features Related to Conscientiousness

The highest correlation (0.44) between Conscientiousness and voice features was found for expert-provided personality scores and mfcc_sma[5]_lpc4. Again, this feature is difficult to explain because it is the fourth linear prediction coefficient of the fifth MFCC Coefficient, which depends on the complex way in which the energy is distributed in different frequency bands.

The highest correlation with self-reported scores (0.30) was with the skewness (which measures the asymmetry of a statistical distribution) of the variations (delta) of the smoothed (sma) variance of the spectral magnitude (pcm_Mag_spectralVariance). Since the correlation is positive, Conscientiousness seems to be related to voices in which the variance of the spectral magnitude increases slower and decreases faster.

For scores reported by close others, the highest correlation was −0.30 with the second Linear Prediction Coefficient of the smoothed Psychoacoustic sharpness. Psychoacoustic sharpness measures how much a sound’s spectrum is in the high end, but the second Linear Prediction Coefficient is related to how much a sample two steps in the past can predict the current value, which is difficult to interpret.

Our results from the first sample seem to indicate that some of the voice characteristics extracted in the features used in the study could be related to the expression of the personality of the individuals. A summary description of the psychological significance of the features found can be seen in Table 5.

#### 3.1.6. Comparison with Results Obtained in a Different Set of Subjects

To analyze the degree to which the previous results were generalizable, we repeated the correlational analysis between the features extracted from the voice analysis and the personality self-assessment in the second set of subjects. This analysis showed that the feature most closely associated with Neuroticism was mfcc_sma[1]_percentile99.0 (r = 0.52; *p* < 0.001); with Extraversion was F0final_sma_quartile3 (r = 0.46; *p* = 0.002); with Openness to Experience was mfcc_sma[3]_kurtosis (r = 0.52; *p* < 0.001); with Agreeableness was mfcc_sma[2]_skewness (r = −0.46; *p* = 0.002); and with Conscientiousness was mfcc_sma_de[7]_kurtosis (r = −0.53; *p* < 0.001).

The results obtained in both samples did not coincide. We must note that we compared the acoustic feature with the highest correlation among a large set of 1941 features. While we do not expect to find the same feature, we do hope to find somewhat related features (e.g., representing variations in the same acoustic magnitude). However, the results do not show these coincidences either. In the Section 4, we will go back to this issue.

### 3.2. Prediction Analysis

After the correlation analysis, we tried to train a predictive model for each of the dimensions. Due to the relatively small sample of participants, we used a leave-one-out strategy. In total, 100 were trained for each dimension (i.e., Neuroticism, Extraversion, Agreeableness, Openness, and Conscientiousness) using the five features with the highest correlations, training with 99 subjects and testing with the remaining subjects.

The personality trait values were categorized into low/medium/high levels, and a classification model using a random forest was trained to distinguish between those categories. Other models, such as support vector machines (SVMs) and multiple linear regression models were also tested, but random forests consistently outperformed other models. Given that we are interested in evaluating the predictive power of the voice features rather than in the accuracy obtained, we only report results with random forests. Random forest is a method for classification and regression tasks that consists of building multiple decision trees [53] and is particularly robust in contexts with limited data, as in the present study. The random forest models were implemented using the standard Python package sci-kit-learn [54]. The default configuration was used except for the number of estimators, which was modified to 20. The model predicts the value of the dimension and checks which group it corresponds to (if the value is smaller than or equal to the 25th percentile, if the value lies between the 25th and 75th percentiles, or if it is greater than the 75th percentile). The percentiles were calculated using the training data. The analyses were carried out regarding the self-report of the subjects, the evaluation of their close others, and the evaluation of the experts. Table 6 shows the results obtained.

Considering that there is a three-class classification task, choosing the class at random would yield a 33% global accuracy. Therefore, it seems that the voice features provide some information about the different personality traits. However, the central class (between the 25th and 75th percentiles) covered 50% of the samples. Consequently, a model that always predicts the central class would obtain a global accuracy of 50%.

As can be seen, the overall success rate (all cases row) of the estimated prediction models is similar for self-assessment, expert assessment, and close assessment. This global percentage varies between 43% accuracy for Conscientiousness with the self-assessment criterion and 60% accuracy in the Extraversion dimension for the expert assessment. This overall hit rate also seems to show that the expert criterion is better predicted by targets’ voice features than the self-criterion and the close others’ criterion for all dimensions. When the scores came from the expert’s ratings, the model achieved an accuracy higher than 50% in any characteristic. A detailed look at the hit rates in the different trait level groups shows that the prediction model performs best in predicting cases with medium trait levels. In all three criterion conditions and for all five personality dimensions, the highest hit percentage always occurs for this group of cases (except the high cases classified in the Conscientiousness dimension in the close-others assessment criterion: 64.3%). Additionally, it can also be observed that cases categorized as low on the trait are better classified than cases categorized as high on the trait.

## 4. Discussion

According to our results, voice features are related to the externalization of individuals’ personality characteristics. The results have shown that Big Five personality predictions based on speech analysis range from 0.3 to 0.4, with the dimensions Neuroticism and Extraversion showing the highest correlations (0.38 and 0.40, respectively). These results support the idea that speech is part of a set of expressive behaviors that denote the individual’s personality [6,44] and might be the basis upon which people make estimations about the inner characteristics of others [7]. Our results show similar, or even higher, correlation values as those obtained with nonverbal communication patterns [55]. We have also found that voice features predict, at least partially, either the estimations an expert produced about the target individuals’ personality traits after watching recordings of them or the judgments significant others make about their acquaintances. Our prediction results based on voice features are more limited than results obtained from facial expressions analysis. In [56], the authors report over 75% accuracy in the prediction of Openness to Experience, Neuroticism, and Extraversion, compared to our 60% best case based on voice features. In any case, we consider that the prediction of personality traits is a fundamentally multimodal task that requires analysis of voice, image, semantics, and other features.

Judgments about others’ inner characteristics and dispositions are commonly carried out in human interactions and probably play a crucial role in social life [57]. People are fairly accurate in making such estimations, as shown by the meta-analysis regarding the accuracy of personality ratings people give to others, even with zero acquaintance [3]. Our predictions are comparable with other people’s accuracy, particularly when comparing the close-others judgments with the target individuals’ own self-report.

Moreover, the results we obtained have shown that the externalization and accuracy facets of a speaker’s voice are related but do not overlap. According to our results, there are coincidences, but also important differences, among the acoustic features that we found more correlated with the personality traits, as measured by the subjects themselves, their relatives, or the experts. It is worth noting that, in general, we found that the largest correlations between the acoustic features and the personality traits were those provided by an expert who was not acquainted with the subject. Interestingly, in these cases, judges based their estimations purely on the video and audio evidence without other important information, such as knowledge about the past behavior of the subjects or the expression of a self-concept the target might have produced to acquaintances.

However, our results and those obtained in some other studies [25] also show there is a lack of agreement between which exact features are related to which specific personality traits. This lack of agreement can be explained by the large number of acoustic features that have been used in the current piece of research. In this study, we analyzed up to 1941 features. In fact, the large number of features makes an exact statistical coincidence unlikely. This, together with the relatively small number of subjects usually included in the studies (100 in our case), may reduce the robustness of the estimations. Nevertheless, there are other factors always present in the speech signal that may also affect the robustness of the extraction of the specific voice features. These include noise and other confounding variables such as emotional states, lexical content, or the speaker’s physical characteristics.

In any case, our results match previous studies that showed a relationship between speech and personality [7,8,10,13]. For instance, extroverted people were prone to talk louder, showed fewer pauses and hesitations, and had higher speech rates [8,13].

More recently, Mairesse et al. [14] found that differences in intensity (the variation and mean) were related to Extraversion, and people scoring higher in Neuroticism tend to present lower and constant voice intensity. These results are partially in agreement with ours. We found that variations in different frequency bands’ energy, which is related to variations in voice volume, were related to Extraversion. We also found that the shimmer of the voice was related to Neuroticism. However, we found that the irregularities in the distribution of the variations (probably due to instabilities in the speech production), instead of constant voice intensity, predicted Neuroticism. Both results are compatible with the expressive characteristics of either Extraversion or Neuroticism, respectively.

Variations in voice volume also correlated with the expert’s ratings of others’ Extraversion (−0.50). It should be noted that the estimations of others’ Extraversion showed the highest accuracy among the Big Five dimensions. In other words, variations in voice volume might be the basis of such estimations, whereas significant others based their estimations on how fast the target spoke, which, for this feature, was less accurate than those used by the expert.

The close others’ estimations showed significant correlations with voice features proceeding from the same cluster as the target individuals in Neuroticism (Shimmer), Openness (Higher voicing estimation), Conscientiousness (energy in lower frequencies), and Agreeableness (dispersion in the speed after a pause).

However, the contribution of this work goes beyond the identification of the voice features that are associated with different personality traits. Most previous studies have used either written texts or limited voice productions, usually collected in situations that are far from natural, e.g., reading texts. They have also used actors representing different personalities. Similarly, they are based on the analysis of an essay corpus consisting of a set of words and word categories based on networks of the Systemic Functional Grammar Theory [14,58,59]. This study aimed to test whether automatic voice analysis of data gathered in a controlled assessment situation was able to estimate the self-reported Big Five personality traits. Based on our findings, there are several practical implications for real-world contexts, particularly in areas such as personnel selection, professional development, and social interactions.

First, the correlations found between voice features and personality traits, especially Extraversion and Neuroticism, suggest that voice analysis could be an effective tool for personality assessment in professional settings (e.g., in job interviews). For instance, it is well known that recruiters may fall into different attribution biases in job interviews due to paying attention to externalized cues not related to the appraised dimensions (e.g., personal attractiveness has been usually associated with positive and favorable traits; see, for instance, [60]). Automatic personality assessment could help recruiters double-check their judgments and detect their biases. Organizations could potentially integrate automatic voice analysis into their assessment protocols to complement traditional evaluations, enhancing the accuracy of personality trait estimations and improving decision-making in personnel selection processes.

Additionally, our results highlight the importance of understanding the nuances in voice-related cues, particularly in social contexts where individuals make judgments about others’ personality traits based on speech. Given that voice features showed higher correlations with non-acquainted experts judging target individuals’ personality traits compared to close others’ judgments, it is recommended that such tools be carefully calibrated to reduce bias stemming from personal acquaintance. Furthermore, as certain voice features (e.g., shimmer and volume variations) have shown moderate predictive power, automated systems could be refined to handle diverse emotional states and physical characteristics, which may otherwise introduce variability in estimations. Overall, these advancements could contribute to more objective scalable assessments in both formal and informal settings while ensuring that individual differences are considered.

Nonetheless, the lack of consistency among studies regarding the specific voice features associated with the different personality characteristics also demands future investigation before this technology can be administered in natural settings. In this vein, an effort to improve the interpretability of these features should be at the core of the future studies’ aims, For this purpose, a viable strategy, similar to lexical approaches to the study of personality, may consist of reducing the dimensionality of the large number of features currently used in the different corpora into factors that can be more easily interpreted. The combination of the two approaches [27] that have separately addressed the topic, one coming from the research on artificial intelligence (AI), big data analysis, and personality computing, where a large number of features are required to maximize prediction and the study of personality traits and personality judgments where psychological meaningfulness of the features is highlighted, must govern further research.

This study is not without its limitations. Firstly, the participants consisted of psychology students. This means that the group was homogeneous not only in age but also probably in personality normalcy. Therefore, it is unlikely to have included individuals with extreme values in the dimensions related to abnormal personality. Furthermore, the homogeneity of the sample presents challenges in terms of generalizing the findings to more diverse populations. Since the participants share similar age and personality profiles, the results may not apply to individuals with extreme or atypical personality traits. This limitation can impact the external validity of the study. Additionally, the strength of the correlations observed between voice features and personality traits should be interpreted cautiously, as the effect sizes, although moderate, may not hold in more varied or larger populations. Future studies with more heterogeneous samples could provide more robust conclusions.

In addition, to address the potential influence of cultural variables on our findings, we recognize that future research should consider expanding the sample to include a more culturally diverse population. Cultural norms and communication styles can influence vocal expressiveness and may affect how personality traits are externalized through voice features. By incorporating individuals from varied sociocultural contexts, future studies could explore whether the correlations found between specific acoustic parameters and personality traits (e.g., Extraversion and Neuroticism) hold across different cultures or if they are context-dependent. This would enhance the generalizability of the predictive models and provide deeper insights into the interaction between culture and vocal cues in personality assessment.

Secondly, we must admit that confounding or latent variables could have been inadvertently included in our study, consequently leading to biased results. For instance, some audio files contained higher noise levels, which could potentially alter some of the voice features found to be correlated with personality dimensions. Moreover, two different microphones were used for collecting the data for samples 1 and 2, respectively, which may have also contributed to the lack of consistency between both samples. Likewise, there is a plethora of factors that can influence a person’s voice, e.g., suffering from a cold, fatigue, emotional states, etc., all of which could potentially act as latent variables, confounding the relationship between voice and personality dimensions. Therefore, these results need to be confirmed by future experiments with either more similar data or with several of the corpora already available for the study of personality trait assessment. In any case, the feasibility of automatic personality systems based on voice features and some other expressive behaviors should be able to deal with suboptimal data gathering such as noise, heterogeneity of recording devices, and particular individual voice characteristics and internal states. The range of possible confounding factors is still very large and should be studied and analyzed in the future as a possible reason for the non-coincident results of different studies. For example, in [61], the authors found that the correlations between voice features and personality traits are highly dependent on the speech task requested. While in our study the task is controlled, we combine the voice gathered in a few different tasks rather than analyzing them as separate tasks.

Finally, the study has only focused on the vocal aspects of the voice. According to Scherer [8], there are other aspects of the ‘externalization of language’, including fluency aspects of speech style, i.e., pauses and discontinuities; morphological and syntactical aspects; and conversational behavior. Future research must incorporate an analysis of all these aspects and face the challenge of including them in such a useful way to contribute to the development of an automatic personality assessment system. In this vein, Breil et al.’s [24] review stressed the role of some other nonverbal cues, such as facial expression or body language, and some other studies have shown how movement can also predict personality appraisals [62].

## Figures and Tables

**Table 1:** LLD low-level descriptors.

Low-Level Descriptor	Description and Interpretation
Loudness	It is a measure of subjective perception of sound pressure.
Zero crossing rate	It is the number of times the amplitude crosses the zero value in a given interval.
Psychoacoustic sharpness	It is how much a sound’s spectrum is in the high end.
Harmonicity	It represents the degree of acoustic periodicity. An HNR of 0 dB means that there is equal energy in the harmonics and in the noise.
MFCC 1–10	Mel Frequency Cepstral Coefficients are a representation of the short-term power spectrum of a signal in a psychoacoustic (Mel) scale.
Kurtosis	It is a statistical measure that is used to describe the distribution of a signal, it measures extreme values in tail relative to a normal distribution. High kurtosis has heavy tails or outliers and low kurtosis has light tails or lack of outliers.
Skewness	It is a statistical measure that is used to describe symmetry. Skewness near zero means symmetric data.
Jitter and Shimmer	Both represent the variations in vibration of the vocal chords. Jitter is the variability in frequency and shimmer is the variability in amplitude.
Spectral Flux	It is a measure of how quickly the power spectrum of a signal is changing.
Voicing	Vibration of the vocal chords.

**Table 2:** Set of 42 functionals.

Functional	Description
Statistical functionals (23)	Arithmetic mean (1), root quadratic mean (2), standard deviation (3), flatness (4), skewness (5), kurtosis (6), quartiles (7–9), inter-quartile ranges (10–12), 1% percentile (13), 99% percentile (14), percentile range 1–99% (15), percentage of frames where the contour is above the minimum + 25% (16), 50% (17) and 90% (18) of the range, percentage of frames where contour is rising (19), maximum (20), mean (21), minimum (22) and standard deviation of segment length (23).
Regression functionals (4)	Linear regression slope (1) and corresponding error (2), quadratic regression coefficient (3) and error (4).
Local minima/maxima related functionals (9)	Mean and standard deviation of rising and falling slopes (1–4), mean (5) and standard deviation (6) of inter maxima distances, amplitude mean of maxima (7), amplitude range of maxima (8) and minima (9).
Others (6)	Linear Prediction Coefficients (LPC) (1 to 5), Linear Prediction (LP) gain (6)

**Table 3:** Descriptives and inter-correlations between personality measures.

		M	SD	Alpha	2	3	4	5	6	7	8	9	10	11	12	13	14	15
1	Self-N	32.89	7.65	0.83	−0.25 * (0.011)	−0.17 (0.076)	−0.24 * (0.017)	−0.27 ** (0.005)	0.32 ** (0.001)	−0.13 (0.200)	−0.02 (0.828)	−0.05 (0.583)	−0.06 (0.537)	0.19 (0.057)	−0.14 (0.168)	−0.09 (0.367)	0.10 (0.316)	−0.19 (0.060)
2	Self-E	45.01	7.32	0.87		0.22 * (0.024)	0.15 (0.134)	0.10 (0.332)	0.11 (0.289)	0.51 ** (<0.001)	0.03 (0.789)	−0.07 (0.477)	−0.09 (0.373)	−0.14 (0.170)	0.31 ** (0.001)	0.10 (0.297)	0.03 (0.770)	0.05 (0.581)
3	Self-O	43.52	8.32	0.87			0.09 (0.354)	0.11 (0.276)	0.08 (0.419)	−0.07 (0.459)	0.13 (0.176)	0.09 (0.370)	0.013 (0.899)	−0.15 (0.142)	−0.10 (0.312)	0.14 (0.150)	−0.07 (0.451)	0.19 (0.090)
4	Self-A	40.96	6.69	0.77				0.37 ** (<0.001)	0.11 (0.258)	−0.02 (0.835)	0.01 (0.892)	0.36 ** (<0.001)	−0.05 (0.594)	0.13 (0.192)	−0.15 (0.130)	0.07 (0.461)	0.14 (0.150)	0.27 * (0.022)
5	Self-C	43.57	8.02	0.88					0.01 (0.959)	0.08 (0.422)	0.03 (0.730)	0.00 (0.966)	0.41 ** (<0.001)	0.00 (0.966)	−0.08 (0.396)	0.01 (0.929)	0.21 * (0.035)	0.34 ** (<0.001)
6	Rel-N	1.92	0.67	S.I.						−0.07 (0.467)	−0.13 (0.210)	−0.05 (0.601)	0.02 (0.809)	0.17 (0.093)	−0.06 (0.537)	0.07 (0.170)	0.16 (0.278)	0.04 (0.097)
7	Rel-E	2.32	0.69	S.I.							0.15 (0.127)	−0.04 (0.665)	−0.08 (0.411)	−0.06 (0.524)	0.27 ** (0.007)	0.13 (0.188)	−0.13 (0.217)	−0.18 (0.066)
8	Rel-O	2.30	0.66	S.I.								0.16 (0.120)	0.04 (0.711)	0.07 (0.464)	0.03 (0.753)	0.16 (0.101)	0.08 (0.407)	−0.12 (0.218)
9	Rel-A	2.04	0.76	S.I.									0.06 (0.520)	0.15 (0.121)	0.16 (0.119)	0.15 (0.138)	0.04 (0.683)	0.25 * (0.010)
10	Rel-C	2.50	0.61	S.I.										0.04 (0.675)	0.07 (0.492)	0.00 (0.985)	0.17 (0.086)	0.27 ** (0.006)
11	Exp-N	8.53	3.46	S.I.											−0.38 ** (<0.001)	−0.23 * (0.017)	0.35 ** (<0.001)	−0.21 * (0.032)
12	Exp-E	7.80	3.71	S.I.												0.48 ** (<0.001)	−0.07 (0.464)	−0.10 (0.298)
13	Exp-O	6.91	3.79	S.I.													−0.13 (0.204)	0.04 (0.715)
14	Exp-A	8.37	3.17	S.I.														0.10 (0.314)
15	Exp-C	8.22	3.52	S.I.														

Note: ** *p* < 0.01; * *p* < 0.05; *p*-value between brackets; M, Mean; SD, Standard deviation; S.I., Single item (no reliability estimation); Self-N, Self-Neuroticism; Self-E, Self-Extraversion; Self-O, Self-Open-to-Experience; Self-A, Self-Agreeableness; Self-C, Self-Conscientiousness; Rel-N, Relative-Neuroticism; Rel-E, Relative-Extraversion; Rel-O, Relative-Openness; Rel-A, Relative-Agreeableness; Rel-C, Relative-Conscientiousness; Exp-N, Expert-Neuroticism; Exp-E, Expert-Extraversion; Exp-O, Expert-Openness; Exp-A, Expert-Agreeableness; Exp-C, Expert-Conscientiousness.

**Table 4:** Voice features with the highest correlation with the personality traits of the subjects, according to (a) self-evaluation, (b) close others, and (c) experts.

	Self (Sample 1)		Close Others (Sample 1)		Experts (Sample 1)	
Trait	Feature	Correlation (*p*-Value)	Feature	Correlation (*p*-Value)	Feature	Correlation (*p*-Value)
Neuroticism	voicingFinalUnclipped_sma_de_percentile1.0	0.37 *** (<0.001)	mfcc_sma[3]_minRangeRel	0.32 ** (0.004)	pcm_Mag_spectralRollOff75.0_sma_quartile1	−0.47 *** (<0.001)
Extraversion	mfcc_sma[3]_lpgain	0.47 *** (<0.001)	voicingFinalUnclipped_sma_de_iqr1-3	0.40 *** (<0.001)	audspec_lengthL1norm_sma_iqr2-3	0.48 *** (<0.001)
Open to Experience	audspec_lengthL1norm_sma_de_upleveltime25	0.36 ** (0.0013)	F0final_sma_de_upleveltime50	−0.41 *** (<0.001)	mfcc_sma[10]_lpc3	−0.36 ** (0.0011)
Agreeableness	audspec_lengthL1norm_sma_de_meanSegLen	−0.33 ** (0.004)	mfcc_sma[5]_risetime	−0.41 *** (<0.001)	pcm_Mag_spectralRollOff75.0_sma_linregc1	0.35 ** (0.002)
Conscientiousness	pcm_Mag_spectralVariance_sma_de_skewness	0.30 ** (0.008)	pcm_Mag_psySharpness_sma_lpc2	−0.30 ** (0.007)	mfcc_sma[5]_lpc4	0.44 *** (<0.001)

Note: *** *p* < 0.001 ** *p* < 0.01; *p*-value between brackets.

**Table 5:** Summary of the psychological meaning of findings from the first sample.

Trait	Summary
Neuroticism	Neuroticism voice features that seem to be related to either instability in voicing or the distribution of energy more biased toward lower frequencies, which could be related to lower volume speech production. Neuroticism is associated with parameters, indicating that most energy is concentrated in low energy levels and could be related to very low voice volume. Additionally, in people with high levels of neuroticism, the smallest variations in the probability of voicing tend to be relatively large, which could indicate instabilities in the production of voiced speech.
Extraversion	Extraversion is related to voice features that seem to be related to longer and more intense pronunciation of vowels and high-energy phones and also to larger variations in voice volume and speaking rate. Features related to Extraversion measure the predictability of the energy in different frequency bands based on recent past values, which is higher when high-energy phonemes, such as vowels, are prolonged and exhibit high energy. Additionally, greater variation in loudness appears to indicate that a larger range of variations in voice volume correlates with higher levels of Extraversion.
Open to Experience	Open to Experience people are related to voice features that are related to either particular intonations (faster increases and slower decreases in pitch) or to having significant variations in voice volume for longer periods of time.
Agreeableness	Agreeableness is related to voice features such as the raising slopes of the spectral roll-off, which implies rapid increases in high frequency energy in the voice. In general, voice features related to Agreeableness seem to be related with changes in the voice volume or frequency distribution, perhaps representing a more variable voice.
Conscientiousness	Conscientiousness presents higher correlation with voice features that are difficult to interpret. It is also the personality dimension with the lowest maximum correlations (with the exception of the correlation with the expert-provided personality scores).

**Table 6:** Accuracy (in percentage) per personality dimension according to the evaluation of self-report, expert, and close others using the five first features with the highest correlation for training.

	All Cases	Low Trait Cases	Medium Trait Cases	High Trait Cases
Self-asessment				
Neuroticism	50	34.5	61.2	44.0
Extraversion	46	37.0	62.7	18.2
Open to Experience	46	35.7	65.3	17.4
Agreeableness	53	46.1	72.5	17.4
Conscientiousness	43	23.1	72.0	4.1
Expert-assessment				
Neuroticism	57	42.8	81.1	10.5
Extraversion	60	48.3	76.4	25
Open to Experience	57	32	79.2	31.2
Agreeableness	56	43.7	78.4	11.8
Conscientiousness	53	57.7	70.0	12.5
Close Others-assessment				
Neuroticism	54	29.6	76.4	22.2
Extraversion	57	15.4	65.1	61.4
Open to Experience	45	18.2	47.9	48.8
Agreeableness	52	53.8	52.4	50.0
Conscientiousness	54	0	47.4	64.3

## Data Availability

Data are available upon request due to privacy restrictions.

## Supplementary Materials

The following supporting information can be downloaded at: https://www.mdpi.com/article/10.3390/s24227151/s1, Close-others judgments on target individuals’ personality assessment rubric.
