# Supplementary material for: Feasibility of Big Data Analytics to Assess Personality Based on Voice Analysis
Source: Sensors (Basel). 2024 Nov 7;24(22):7151. doi: 10.3390/s24227151 (PMC11598682; doi:10.3390/s24227151)
Supplement: Supplementary file 1 [file sensors-24-07151-s001.zip › sensors-3262418-supplementary.pdf]

Supplementary material S1
Close-others judgments on target individuals' personality assessment rubric

Rubric for relative/close other judgment of target individual's personality.

Instructions:
The table below includes three different descriptions of people in each row. Please, choose one of the three options in each row which best describes your acquaintance, and circle your response.

Your acquaintance is:

Safe, resistant and generally relaxed, even in stressful situations.	Generally calm and able to face stressful situations. But, sometimes experiences feelings of guilt, anger or sadness.	Sensitive, emotional and prone to experiencing unpleasant sensations.

Introverted, reserved and serious. They prefer to be alone or in the company of very close friends.	Moderate in terms of activity and enthusiasm. They appreciate the company of others, but also enjoy solitude.	Extraverted, open, active and energetic. They like to be surrounded by people.

With their feet on the ground and practical. Traditional and committed to existing methods.	Practical and at the same time eager to try new ways of doing things. Finds a balance between the new and the old.	Open to new experiences. Has a wide range of interests and is very imaginative.

Realistic, skeptical, proud and competitive. They tend to express anger with little regard for others.	Generally nice, warm and quiet. But sometimes can be stubborn and competitive.	Compassionate, sensitive and willing to cooperate and avoid conflicts.

They do not organize well and sometimes show little care in their work. They prefer not to make plans.	Formal and moderately well organized. They usually have clear objectives, but are also able to put aside their work.	Responsible and organized. Has solid principles and does not stop until their objectives have been reached.

Supplementary Material S2

Expert's ratings of target individual's personality sheet

Instructions:

Please, rate the target's Big Five personality dimensions with reference to the definitions and characteristics of each of them and place a mark in the gray bar according to the estimation.

Neuroticism: Anxious, nervous, fearful, insecure, frightened **vs**. Calm, relaxed, calm, balanced.

Typical behavioral aspects of Neuroticism: non-verbal nervousness, verbal uncertainty, negative comments about themselves, tension in facial or body expression, little fluency in verbal expression, use of words related to anxiety and

Safe, resistant and generally relaxed, even in stressful situations.	Generally calm and able to face stressful situations. But, sometimes experiences feelings of guilt, anger or sadness.	Sensitive, emotional and prone to experiencing unpleasant sensations.

Extraversion: sociable, talkative, active, impulsive, extroverted **vs.** timid, reticent, passive, deliberative, reserved.

Typical behavioral aspects of Extraversion: expressive verbal and non-verbal behavior, impulsive manifestations, striking appearance, social contact seeking, expressive facial expression, expressive and no low tone of voice, amplitude in verbal explanations, references to others in speech, elegant or ostentatious dress style.

Introverted, reserved and serious. They prefer to be alone or in the company of very close friends.	Moderate in terms of activity and enthusiasm. They appreciate the company of others, but also enjoy solitude.	Extraverted, open, active and energetic. They like to be surrounded by people.

Open to Experience: imaginative, civilized, educated, interested, gifted **vs.** unimaginative, primitive, rude, indifferent, intellectually limited.

Typical behavioral aspects of Open to Experience: competent intellectually, with creative and original ideas, open to unusual situations, verbal eloquence, original and unusual stories, breadth of the story told.

With their feet on the ground and practical. Traditional and committed to the existing methods.	Practical and at the same time eager to try new ways of doing things. Finds a balance between the new and the old.	Open to new experiences. They have a wide range of interests and are very imaginative.

Agreeableness: Confident, friendly, helpful, well-meaning, good feelings **vs.** obstinate, quarrelsome, hard-hearted, resentful.

Typical behavioral aspects of Agreeableness: helpful, friendly and trustworthy verbal and nonverbal behavior, obedient, non-aggressive, disinterested comments and social orientation, uses a friendly voice in the interview, uses words referring to the family or social processes, does not use aggressive words, in the situation of having to wait does not get aggressive and impatient, shows a careful posture in speaking and presentation.

Realistic, skeptical, proud and competitive. They tend to express their anger with little regard.	Generally nice, warm and quiet. But sometimes can be stubborn and competitive.	Compassionate, sensitive and willing to cooperate and avoid conflicts.

Conscientiousness: meticulous, reliable, orderly, demanding, meticulous **vs.** careless, unreliable, chaotic, frivolous, erratic.

Typical behavioral aspects of Conscientiousness: Committed to common agreements, correct linguistically, avoids words or misconceptions, appearance formal, understandable and clear verbal expression.

They do not organize well and sometimes shows little care in their work. They prefer not to make plans.	Formal and moderately well organized. They usually have clear objectives, but are also able to put aside their work.	Responsible and organized. They have solid principles and do not stop until their objectives have been reached.
